# Prospective Observational Multisite Study of Handover in the Emergency Department: Theory versus Practice

**DOI:** 10.5811/westjem.2020.9.47836

**Published:** 2021-01-12

**Authors:** Philipp Ehlers, Matthias Seidel, Sylvia Schacher, Martin Pin, Rolf Fimmers, Monika Kogej, Ingo Gräff

**Affiliations:** * University Hospital Bonn, Department of Emergency Medicine, Bonn, Germany; † Hospital Köln Kalk, Department of Emergency Medicine, Cologne, Germany; ‡ Hospital Florence Nightingale Düsseldorf, Department of Emergency Medicine, Düsseldorf, Germany; § University Hospital Bonn, Institute for Medical Biometry, Informatics and Epidemiology, Bonn, Germany

## Abstract

**Introduction:**

The handover process in the emergency department (ED) is relevant for patient outcomes and lays the foundation for adequate patient care. The aim of this study was to examine the current prehospital to ED handover practice with regard to content, structure, and scope.

**Methods:**

We carried out a prospective, multicenter observational study using a specifically developed checklist. The steps of the handover process in the ED were documented in relation to qualification of the emergency medical services (EMS) staff, disease severity, injury patterns, and treatment priority.

**Results:**

We documented and evaluated 721 handovers based on the checklist. According to ISBAR (Identification, Situation, Background, Assessment, Recommendation), MIST (Mechanism, Injuries, Signs/Symptoms, Treatment), and BAUM (Situation [German: *Bestand*], Anamnesis, Examination [German: *Untersuchung*], Measures), almost all handovers showed a deficit in structure and scope (99.4%). The age of the patient was reported 339 times (47.0%) at the time of handover. The time of the emergency onset was reported in 272 cases (37.7%). The following vital signs were transferred more frequently for resuscitation room patients than for treatment room patients: blood pressure (BP)/(all comparisons p < 0.05), heart rate (HR), oxygen saturation (SpO_2_) and Glasgow Coma Scale (GCS). Physicians transmitted these vital signs more frequently than paramedics BP, HR, SpO_2_, and GCS. A handover with a complete ABCDE algorithm (Airway, Breathing, Circulation, Disability, Environment/Exposure) took place only 31 times (4.3%). There was a significant difference between the occupational groups (p < 0.05).

**Conclusion:**

Despite many studies on handover standardization, there is a remarkable inconsistency in the transfer of information. A “hand-off bundle” must be created to standardize the handover process, consisting of a uniform mnemonic accompanied by education of staff, training, and an audit process.

## INTRODUCTION

Medical handover from prehospital care to the emergency department (ED) is defined as the transfer of responsibility of the care of one or more patients to another person or team.^1^,^2^ Handovers, especially in the ED, are of enormous significance for the subsequent emergency treatment because that treatment requires precise timing, rapid decision-making, and specific expertise.^2^,^3^ Furthermore, the handover is critical for the relaying of information, such as interventions that have occurred and details from the emergency scene. The transfer from prehospital care to the ED is always an interprofessional process involving at least two professional groups. This can lead to misunderstandings and dissatisfaction due to different expectations and approaches.^2^,^4^–^6^

### Importance

Studies by the Australian Commission on Safety and Quality in Health Care have shown that the quality of handover decreases based on an increasing rate of adverse events due to a lack of structure and communication, particularly in the presence of complex patient problems.^7^ Inadequate, incorrect, or misleading information puts patients at risk.^8^ Inadequate communication was one of the most frequent causes of malpractice claims reported to the Joint Commission between 1995–2006.^9^ A study published in 2016 showed that communication errors caused 1744 deaths and resulted in costs of 1.7 billion US dollars over a period of five years in American hospitals.^10^,^11^ The transmission of information in a stressful, highly dynamic work environment such as the ED represents a high-risk source of treatment errors and avoidable adverse events and is therefore relevant to patient outcomes influencing mortality.^12^–^14^

### Aim

As early as 2007, the Joint Commission called for the process of handover to be standardized with the aim of increasing patient safety.^15^ In 2008, the World Health Organization (WHO) formulated the development of “standard operating procedures” in communication as one of the five priorities in the area of patient safety for industrialized countries.^9^,^16^ To date, a large number of protocols for the standardization of oral handovers have been published.^17^,^18^ In addition to checklists, computer-assisted handover programs and algorithms as well as specific mnemonics have been established to serve as reminders intended to provide guidance when following a process.^19^

The goal of this study was to examine the handover procedures in an ED, focused on content, scope and structure and the application of existing handover mnemonics. Our project was a prospective observational study of the handover process, focusing on the interface between the prehospital care and the ED.

## METHODS

### Emergency Medical Services System Organization

Emergency care in Germany is provided by rescue vehicles that are manned by one of two types of clinicians – paramedics or emergency physicians (EP). The responsible emergency call center decides when an EP is called to the scene according to pre-specified criteria. In many cases, following initial emergency medical care under the supervision of the EP, further transport of the patient is then carried out by the paramedics. In Germany, paramedic training consists of a three-year course with theoretical and practical content as well as a final examination. Physicians can acquire an additional qualification with focus on emergency medicine (EM). This includes 24 months of clinical specialist training, an additional six months of anesthesia, intensive care and EM expertise, an 80-hour theory course, 50 life-saving emergency medical services (EMS) missions, and a final examination. The EMS staff or the responsible control center, respectively, is in charge of the pre-registration of emergency patients at the ED. The above-mentioned training courses historically have had no specific focus on training with regard to the EMS handover process.

**Population Health Research Capsule**

What do we already know about this issue?

The handover process is relevant for adequate treatment of patients and therefore affects patient outcomes. Consequently, it plays a major role in patient safety.

What was the research question?

We sought to provide a current status of handover practice in EDs with regard to content and structure.

What was the major finding of the study?

To date no handover standard has been established and current practice reveals deficits in structure.

How does this improve population health?

This study raises awareness of the need to include handover in national health policy programs, thereby accelerating the process of standardization.

### Design

We carried out a prospective, multicenter observational study. To minimize bias and to allow comprehensive assessment of EMS handovers, we used a checklist. The checklist was derived by including elements from the following established mnemonics, which are benchmarks in the handover literature: ISBAR (Identification, Situation, Background, Assessment, Recommendation), MIST (Mechanism, Injuries, Signs/Symptoms, Treatment) and BAUM (Situation [German: *Bestand*], Anamnesis, Examination [German: *Untersuchung*], Measures).^20^–^23^ The checklist was developed by a selected expert committee of five EPs and paramedics with experience in both prehospital and clinical EM. The final checklist contains all relevant core contents for memory (SAMPLER algorithm – Symptoms, Allergies, Medication, Past medical history, Last oral intake, Events prior to incident, Risk factors) and assessment of the patient’s condition (ABCDE algorithm - Airway, Breathing, Circulation, Disability, Environment/Exposure) as well as vital signs. The ABCDE and SAMPLER algorithms are recommended by WHO for patient treatment according to priority but not specifically for handover process. Both algorithms are core elements of baseline paramedic training and advanced training courses such as International Trauma Life Support (ITLS) and Prehospital Trauma Life Support (PHTLS).^24^,^25^ Detailed descriptions of all mnemonics and algorithms are provided in the electronic attachment.

To gather more nuanced data, handovers were assessed by profession (paramedic vs physician), and severity of the disease/treatment priority (resuscitation vs treatment room), as well as trauma vs non-trauma patients.

### Setting

The study was carried out at three hospitals with different emergency care levels. The University Hospital Bonn (UKB) is a comprehensive care hospital with about 45,000 ED visits per year. The Florence Nightingale Hospital in Düsseldorf sees approximately 37,000 emergency patients per year and is a teaching hospital of the University Hospital Düsseldorf. The Protestant Hospital in Cologne Kalk is a non-tertiary teaching hospital of the University Hospital Cologne that cares for approximately 20,000 ED patients per year. In these three EDs, EMS are not specifically required or trained to use any particular handover structure. In this study, we examined only handovers by EMS paramedics or physicians.

### Data Collection

During the test period from March 11, 2019–October 31,2019, under supervision of the Institute for Medical Biometry, Informatics and Epidemiology of the University Hospital Bonn, study personnel documented the handovers in EDs between 6:30 am – 9:30 pm. To minimize loss of information, details on prehospital care were obtained from the emergency services documentation record. To ensure standardized application of the checklist and to minimize errors in the documentation, only persons directly involved in the development of the checklist carried out the application. Due to limited personnel, not all handovers within the observation period were recorded.

### Statistical Analysis

We used Microsoft Excel 2017 (Microsoft Corporation, Redmond, WA) to manage and tabulate the comprehensive data set. The duration of patient transfer was evaluated descriptively as a continuous variable by specifying the mean value and standard deviation. All other collected data were categorical and were represented by the specification of absolute and relative frequencies, the odds ratio (OR), and the specification of 95% confidence intervals (CI). A statistical comparison of subgroups was carried out using the Chi-square test, or for smaller group sizes, Fisher’s exact test to a significance level of 0.05 (5%). We evaluated all data using SPSS version 26 (SPSS Inc. Chicago, IL).

### Ethics Statement

The study received approval (No. 002/19) from the chairman of the local ethics committee (K. Racké, MD, PhD, Professor, University Bonn). Data obtained from the clinical information system may be used in accordance with the code of medical ethics (article 15/1) (https://www.aekno.de/aerzte/berufsordnung#_15 http://www.aekno.de/page.asp?pageID=57#_15) of the General Medical Council. Furthermore, as stipulated by German data protection regulations, the physician may use existing patient data for analyses without explicitly asking for the consent of patient. All collected clinical data evaluated in this study were fully anonymized prior to analysis. Furthermore, the data collected do not contain any patient information. The study design is consistent with the Declaration of Helsinki.^26^

## RESULTS

### Baseline Characteristics

During the observation period, a total of 721 handovers were examined in the three EDs. Of these handovers, 44.5% (n = 321) were carried out by EPs and 55.5% (n = 400) by paramedics. 79.1% (n = 570) of the transfers invovled non-trauma emergency patients, and 20.9% (n = 151) patients after trauma. Of the transfers, 30.5% (n = 220) took place in the resuscitation room, the remaining 69.5% (n = 501) in normal treatment rooms or in the triage room. The mean value of the transfer time was one minute 11 seconds. (standard time deviation STD ± 0:34 minutes). In 74.5% (n = 537), the ED personnel raised further questions for better understanding.

### Identification, Mechanism and Medical Situation

The sex of the emergency patient was mentioned with a frequency of 95.6% (n = 689) at the time of delivery; the name of the patient was mentioned with a frequency of 83.8% (n = 604) and the age of the patient was mentioned in 47.0% (n = 339) of the cases. The suspected diagnosis was reported in 95.7% (n = 690) and the emergency event in 90.4% (n = 652). Comparatively less frequently, information regarding the place where the emergency occurred was reported in 66.4% (n = 479) and the time it occurred in 37.7% (n = 272) of cases (Table 1).

### Leading Priority and Vital Signs

The frequency of the handovers in which the ABCDE algorithm recommended by WHO was completely applied (chronological mention of all elements) was 4.3 % (n = 31). The subgroup analysis shows that physician staff performed a complete ABCDE handover 7.2% of the time, compared to paramedics who used it 2.0% of the time (OR: 3.8, p < 0.05). Also, the complete ABCDE algorithm was applied to resuscitation room patients more frequently (OR: 7.2, p < 0.05), compared to transfers in the conventional treatment rooms or the triage room. The same trend was observed in the transfer of trauma patients compared to non-trauma emergency patients (OR: 18.7, p < 0.05). In 86.1% (n = 621) of the handovers, the ABCDE algorithm was not applied, while in 3.2% (n = 23), a handover with at least three points of the ABCDE algorithm took place (Table 2).

Looking at the prehospital vital signs and their communication during handover, the following pattern becomes apparent: In only 44.7% (n = 289) of cases was the blood pressure (BP) mentioned in the handover. In 30.6% (n = 199) of handovers the heart rate (HR) was verbalized, while the oxygen saturation (SpO_2_) was only communicated in 25.6% (n = 165) of cases. The respiratory rate was only communicated in 12.8% of handovers. The testing of circulation, sensation and mobility (CSM) was communicated much more often, in 76.9% of cases. Other important elements, such as Glasgow Coma Scale (GCS), blood sugar (BS) and temperature are listed in Table 3.

The subgroup analysis of the different occupation groups shows that trained EPs more often refer to the transmitted vital parameters BP (OR: 1.9), HR (OR: 2.2), SpO_2_ (OR: 2.7) and GCS (OR: 5.1) at the time of handover (Table 4). The subgroup analysis of transfers in resuscitation room patients shows that the above-mentioned vital signs were also more frequently reported compared to handovers in normal treatment rooms (Table 5). Differentiation between trauma patients and non-trauma emergency patients revealed that GCS was mentioned more frequently in trauma patients (p < 0.05).

### Medical History and Risk Factors

Previous illnesses of the emergency patient were reported at the handover with a frequency of 49.7% (95% CI, 46.0–53.3 / n = 358) and the risk factors of the patient in 54.4% (95% CI, 50.7–58.0 / n = 392). The patient’s home medication was mentioned in 41.2% (95% CI, 37.6–44.8 / n = 297) of the cases. Information on existing allergies was significantly less often reported in 17.0% (95% CI, 14.3–19.8 / n = 123) and on the last meal in 3.9% (95% CI, 2.6–5.3 / n = 28) of cases.

In just 1.1% of the cases (n = 8) was the SAMPLER algorithm, recommended by WHO, fully applied (chronological mention of all letters or their contents). In 27.2% (n = 200) of the handovers, at least three contents of the SAMPLER algorithm were mentioned at the handover. The subgroup analysis shows that in comparison to the paramedics, physicians more frequently mentioned at least three SAMPLER components (p < 0.05). The same is true for resuscitation room handovers when compared to the treatment room patients (p < 0.05), and for the trauma vs the non-trauma emergency patients (p < 0.05). In 20.0% (n = 144) of the handovers, no information of the SAMPLER algorithm was transmitted.

### 3.5 Emergency Treatment

Analysis of prehospital therapeutic activities shows the following results: Intravenous (IV) access was mentioned in only 37.2% (n = 132) of the cases at handover and had the lowest ratio between performance and handover of all preclinically performed measures. The preclinically derived 12-lead electrocardiogram was discussed in 75.7% (n = 109) of the cases at handover. In 58.9% (n = 63) of cases, information on prehospital oxygen therapy was provided at the handover. Drug administration and airway management were the most frequently mentioned rescue measures at handover. Defibrillation as a life-saving measure was mentioned in 85.7% of the cases, if performed as a prehospital treatment (Table 6).

The subgroup analysis of resuscitation room patients shows that, in comparison to handover of patients in normal treatment rooms, all prehospital therapeutic measures were mentioned with the same frequency. The only significant difference was found in the establishing of an IV access (p < 0.05).

## DISCUSSION

This is the first prospective study to examine the EMS handover process in German EDs in terms of content, scope, and structure in relation to existing handover mnemonics. The work is intended to present the current handover practice and demonstrates that the handover does not follow a clear protocol and that a pronounced inconsistency exists in information transfer. In addition, differences in the extent and completeness of the handovers are apparent depending on staff and the priority of treatment (resuscitation room vs treatment room) and the injury pattern (trauma vs non-trauma patients). The data collected from the three EDs refer to a large supply area of the rescue service in the German federal state of North Rhine-Westphalia (NRW). Since NRW is the federal state with the highest population in Germany (approximately 18 million) and the structure of emergency services does not differ significantly from that of the other regions of Germany, we believe that the data presented have a high scientific validity for Germany and may have important implications for other countries as well.

The data of the present study are supported by another European study conducted by Delupis et al in Italy. They found comparable results in their work: the absence of standardization of the handover process; a high variability in information transfer; and deficiencies in the transfer of responsibility of patient care.^27^

It is notable that the presence of a higher disease severity with pathological vital signs appears to be a trigger for more verbalization at the handover. Conversely, in less critical patients, information regarding the leading medical problem, vital signs, and other information from the patient’s medical history may not be considered relevant for the handover. To date, numerous studies have shown that vital signs, especially respiratory rate, BP, and GCS, have a predictive value for the outcome of critical emergency patients.^28^,^29^ In this context, vital signs play an important role in order to evaluate critical conditions of patients by using scores such as CRB 65 and qSOFA.^30^,^31^ Here, a transfer of vital signs is categorically called for, independent of the severity of the illness and the qualification of the person transmitting the data. Information on the time component of the emergency event is essential regarding time-critical therapeutic measures including thrombolytics for stroke or time-sensitive sepsis bundles.^32^

The main findings show that with regard to MIST, ISBAR and BAUM, no mnemonics were applied during handover, resulting in a lack of structure and information transfer. This is supported by the high demand for additional information from the receiving team. One explanation lies in the individual design of the handover process, resulting in incongruence between expected and actually transferred information. In our opinion, this is not due to a lack of handover mnemonics, but rather to the fact that to date, no handover practice exists that fully meets the high requirements of a transfer in the ED. According to Nasarwanji et al, not all information necessary for the transfer can be accommodated in a generally valid mnemonic.^33^ Hence, the handover process needs a specifically adapted mnemonic, with elements from the ABCDE or SAMPLER algorithms. Since the handover is strongly influenced by human factors, consideration should be given to integrating crew resource management aspects into the handover process to improve patient safety.^34^ Other handover practices to promote effective transfer of information include the following: no actions performed on patients during the handover; face-to-face communication; presence of all team members; a repeat back of essential handover content; and an opportunity for questions.

This thesis is supported by the work of Keebler et al, who with the help of a systematic literature review and a series of meta-analyses, examined many publications on handover standardization. Keebler et al took on the standardization of the handover in 2017, as called for by the Joint Commission in 2007, and found that all studies follow different standards, enabling only limited comparability.^19^ In their conclusion, the authors recommended that protocols should standardize the handover and provide users with orientation as to what information should be transmitted.

It becomes clear that despite the available mnemonics and the numerous studies on standardization of the handover, we still have a gap between the theoretical handover approach and its practical implementation. The target must be the creation of a shared mental model between emergency services and hospital staff. This would enable handovers in an interprofessional, team-based manner.^35^,^36^ Therefore, future research should concentrate on combining elements of clinical effectiveness and implementation using hybrid study designs to enhance the practical application of specifically adapted mnemonics.^37^ In concrete terms this means developing a mnemonic with the requirements described above, which then is validated using the Delphi method. Subsequently, the effectiveness of the mnemonic and its implementation (ie. its acceptance by paramedics) has to be examined by prospective studys.

Furthermore, national initiatives for the general implementation of handover approaches in the clinical setting are necessary for Germany and other countries, in line with the initiatives already taken in Australia, Great Britain, and the USA. The provision of appropriate financial and human resources for the implementation of this health policy objective is an indispensable prerequisite. In the near future, external audits must review the introduction and application of structured handover processes in relation to triage in the ED. It also seems necessary to include the topic of handover as training content in the curricula of the proven prehospital and hospital course concepts such as Advanced Life Support, ITLS, PHTLS, Advanced Trauma Care for Nurses, and Advanced Trauma Life Support. The handover should be incorporated into the training of paramedics, as well as into the further training programs for EPs and nurses.

## LIMITATIONS

It is possible that this study includes repetitive handovers by the same EMS staff during the observation period. Thus, some of our results may have been limited by our sample population. However, given the large catchment area of the three EDs, this is unlikely to affect the overall significance and results of the study. Additionally, the selected period from March 2019–October 2019 did not allow any conclusions to be drawn for an entire year, as possible seasonal fluctuations were not considered. Furthermore, patient transfers during night shifts were not documented. It cannot be ruled out that the content and scope of the handover may vary regarding the time of day.

It is possible that while applying the checklist, information may not have been recorded or missed. However, we consider the percentage as negligible, since the person documenting never participated in direct patient care and was as an external observer. Finally, since it could not be avoided that several handovers took place at the same time, the external observers were not able to record the data of all handovers in the given observation period. Therefore, it must be assumed that in comparison to the results, both better structured as well as worse structured handovers were not recorded. Nevertheless, due to the high number of cases and the observation at three EDs, the present results create a representative picture of the current handover process.

## CONCLUSION

The present study shows that despite many existing handover protocols, there is no widespread implementation or acceptance of these protocols. Not even the measures recommended by the World Health Organization to increase patient safety are reliably transmitted during handover. Future research should aim at establishing appropriate user-friendly handover protocols for the ED. Improving and standardizing the EMS-to-ED handover process has a high potential to improve patient safety and emergency care.

## Figures and Tables

**Table 1:** Absolute frequency, 95% confidence interval, and evaluated numbers related to treatment location and professional qualification in terms of identification (name, sex, age) and details of emergency event.

	Absolute frequency (n = 721)	Percentage	Resuscitation room (n = 220)	95% CI	Treatment room (n = 501)	95% CI	Physician staff (n = 321)	95% CI	Paramedical staff (n =400)	95% CI
Name	604	83.8%	204 (92.7%)	89.3 – 96.2	400 (79.8%)	76.3 – 83.4	287 (89.4%)	86.0 – 92.8	317 (79.3%)	75.3 – 83.2
Sex	689	95.6%	217 (98.6%)	97.1 – 100.0	472 (94.2%)	92.2 – 96.3	314 (97.8%)	96.2 – 99.4	375 (93.8%)	91.4 – 96.1
Age	339	47.0%	175 (79.5%)	74.2 – 84.9	164 (32.8%)	29,0 – 37.3	229 (71.3%)	66.4 – 76.3	110 (27.5%)	23.5 – 32.5
Suspected Diagnosis	690	95.7%	213 (96.8%)	94.5 – 99.2	477 (95.2%)	93.3 – 97.1	311 (96.9%)	95.0 – 98.8	379 (94.8%)	92.6 – 96.9
Description of Emergency Event	652	90.4%	217 (98.6%)	97.1 – 100.0	435 (86.8%)	83.9 – 89.8	318 (99.1%)	98.0–100.0	334 (83.5%)	79.8 – 87.1
Location of Emergency Event	479	66.4%	177 (80.5%)	75.2 – 85.7	302 (60.3%)	56.0 – 64.6	261 (81.3%)	77.0–85.6	218 (54.5%)	49.6 – 59.4
Time of Emergency Event	272	37.7%	109 (49.5%)	42.9 – 56.2	163 (32.5%)	28.4 – 36.7	155 (48.3%)	42.8–53.8	117 (29.3%)	24.8 – 33.7

*CI*, confidence interval.

**Table 2:** Application of ABCDE algorithm during the handover process dependent on trauma/non-trauma patients, physician/paramedical staff and resuscitation room/treatment room. Additionally, OR, 95% CI and p-value are displayed to allow comparison.

Application of ABCDE algorithm	Handover of trauma patients (n = 151)	Handover of non-trauma patients (n = 570)	OR	95% CI	P-value
No application of ABCDE algorithm	89 (58.9%)	532 (93.3%)	0.1	0.07 – 0.2	<0.05
Partial application of ABCDE algorithm †	37 (24.5)	32 (5.6%)	5.4	3.3 – 9.1	<0.05
Full application of ABCDE algorithm	25 (16.6%)	6 (1.1%)	18.7	7.5 – 46.4	<0.05

Application of ABCDE algorithm	Handover by physician staff (n = 321)	Handover by paramedical staff (n = 400)	OR	95% CI	p-value

No application of ABCDE algorithm	249 (77.6%)	372 (93.0%)	0.3	0.2 – 0.4	<0.05
Partial application of ABCDE algorithm †	49 (15.2%)	20 (5.0%)	3.4	2.0 – 5.9	<0.05
Full application of ABCDE algorithm	23 (7.2%)	8 (2.0%)	3.8	1.7 – 8.6	<0.05

Application of ABCDE algorithm	Handover resuscitation room (n = 220)	Handover treatment room (n = 501)	OR	95% CI	p-value

No application of ABCDE algorithm	149 (67.7%)	472 (94.2%)	0.1	0.08 – 0.2	<0.05
Partial application of ABCDE algorithm †	48 (21.8%)	21 (4.2%)	6.4	3.7 – 11.0	<0.05
Full application of ABCDE algorithm	23 (10.5%)	8 (1.6%)	7.2	3.2 – 16.4	<0.05

† Using at least one and up to four letters of ABCDE algorithm.

*ABCDE*, Airway, Breathing, Circulation, Disability, Environment/Exposure; *OR*, odds ratio; *CI*, confidence interval

**Table 3:** Frequency of preclinically evaluated vital signs with total occurrence and percentage as well as 95% confidence interval during handover.

Vital signs	Total prehospital evaluation (n = 721)	Handover frequency	Percentage	95% CI
Blood pressure	646	289	44.7%	41.0 – 48.6
Heart rate	650	199	30.6%	27.1 – 34.2
Oxygen saturation	645	165	25.6%	22.2 – 29.0
Respiratory rate	382	49	12.8%	9.2 – 15.9
Glasgow Coma Scale	566	126	22.2%	18.8 – 25.5
Blood sugar	400	98	24.5%	20.3 – 28.7
Temperature	262	62	23.7%	18.5 – 28.8
CSM	255	196	76.9%	71.7 – 82.1

*CI*, confidence interval; *CSM*, circulation, sensation and movement.

**Table 4:** Vital signs in terms of prehospital evaluation and handover frequency depending on professional qualification. Odds ratio (OR), 95% confidence interval (CI), and P-value were used to show statistical correlation. The physician provider was used as reference for the development of the OR.

Vital signs	Prehospital evaluation physician staff (n = 321)	Handover frequency	Prehospital evaluation paramedical staff (n = 400)	Handover frequency	OR	95% CI	P-value
Blood pressure	318 (99.1%)	167 (52.5%)	328 (82.0%)	122 (37.2%)	1.9	1.4 – 2.6	<0.05
Heart rate	319 (99.4%)	125 (39.2%)	331 (82.8%)	74 (22.4%)	2.2	1.6 – 3.2	<0.05
Oxygen saturation	317 (98.8%)	111 (35.0%)	328 (82.0%)	54 (16.5%)	2.7	1.9 – 4.0	<0.05
Respiratory rate	238 (74.1%)	36 (15.1%)	144 (36.0%)	13 (9.0%)	2.0	1.0 – 3.9	0.052
Glasgow Coma Scale	294 (91.6%)	100 (34.0%)	272 (68.0%)	26 (9.6%)	5.1	3.2 – 8.2	<0.05
Blood sugar	234 (72.9%)	50 (21.4%)	166 (41.5%)	48 (28.9%)	0.7	0.4 – 1.1	0.084
Temperature	157 (48.9%)	33 (21.0%)	105 (26.3%)	29 (27.6%)	0.7	0.4 – 1.2	0.22
CSM	154 (48.0%)	121 (78.6%)	101 (25.3%)	75 (74.3%)	1.3	0.7 – 2.3	0.42

*CSM*, circulation, sensation and movement.

**Table 5:** Vital signs in terms of prehospital evaluation and handover frequency depending on treatment localization. The resuscitation room was used as reference for the development of the odds ratio (OR), 95% confidence interval (CI), and p-value were used to show statistical correlation.

Vital signs	Prehospital evaluation resuscitation room (n = 220)	Handover frequency	Prehospital evaluation treatment room (n = 501)	Handover frequency	OR	95% CI	P-value
Blood pressure	218 (99.1%)	121 (55.5%)	428 (85.4%)	168 (39.3%)	1.9	1.4 – 2.7	<0.05
Heart rate	219 (99.5%)	81 (37.0%)	431 (86.0%)	118 (27.4%)	1.6	1.1 – 2.2	<0.05
Oxygen saturation	218 (99.1%)	76 (34.9%)	427 (85.2%)	89 (20.8%)	2.0	1.4 – 2.9	<0.05
Respiratory rate	156 (70.9%)	22 (14.1%)	226 (45.1%)	27 (11.9%)	1.1	0.6 – 2.1	0.66
Glasgow Coma Scale	204 (92.7%)	83 (40.7%)	362 (72.3%)	43 (11.9%)	5.0	3.3 – 7.6	<0.05
Blood sugar	170 (77.3%)	40 (23.5%)	230 (45.9%)	58 (25.2%)	0.9	0.6 – 1.4	0.70
Temperature	108 (49.1%)	22 (20.4%)	154 (30.7%)	40 (26.0%)	0.7	0.4 – 1.3	0.29
CSM	131 (59.5%)	106 (80.9%)	124 (24.8%)	90 (72.6%)	1.6	0.9 – 2.9	0.12

*CSM*, circulation, sensation and movement.

**Table 6:** Frequency of prehospital applied treatment with total occurrence and percentage as well as 95% confidence interval during handover.

Prehospital treatment	Prehospital treatment (n = 721)	Handover frequency	Percentage	95% CI
12-channel electrocardiogram	144	109	75.7%	68.6 – 82.8
Oxygen application	107	63	58.9%	49.4 – 68.4
Intravenous access	355	132	37.2%	32.1 – 42.2
Drug administration	295	259	87.8%	34.0 – 91.6
Wound care	29	15	51.7%	32.4 – 71.1
Airway management	41	37	90.2%	80.8 – 99.7
Immobilization	85	42	49.4%	38.6 – 60.3
Defibrillation	7	6	85.7%	50.7 – 100.0

*CI*, confidence interval.
